# Isolating high-quality RNA for RNA-Seq from 10-year-old blood samples

**DOI:** 10.1038/s41598-024-80287-4

**Published:** 2024-12-28

**Authors:** Charlene Portelli, Elisa Seria, Ritienne Attard, Mitra Barzine, Eva M. Esquinas-Roman, Francesca Borg Carbott, Karen Cassar, Matthew Vella, Brendon P. Scicluna, Jean-Paul Ebejer, Rosienne Farrugia, Stephanie Bezzina Wettinger

**Affiliations:** 1 Department of Applied Biomedical Science, Faculty of Health Sciences, University of Malta, Msida, 2080 MSD Malta; 2 Department of Medicine, Faculty of Medicine and Surgery, University of Malta, Msida, 2080 MSD Malta; 3 Centre for Molecular Medicine and Biobanking, University of Malta, Msida, 2080 MSD Malta

**Keywords:** Biobanking, Transcriptomics, Multi-omics, RNA isolation, Long-term storage, RNA-Seq, RNA sequencing, Biological techniques, Gene expression analysis, Isolation, separation and purification

## Abstract

There is much interest in analysing RNA, particularly with RNA Sequencing, across both research and diagnostic domains. However, its inherent instability renders it susceptible to degradation. Given the imperative for RNA integrity in such applications, proper storage and biobanking of blood samples and successful subsequent RNA isolation is essential to guarantee optimal integrity for downstream analyses. Especially for larger collections, it would be particularly beneficial if these methods would additionally offer affordability, minimal blood volume requirements and also long-term storage. In this study, RNA of high quality, suitable for transcriptomics, has been successfully isolated from 400 µL of EDTA and citrated whole blood samples in Boom’s lysis buffer stored at −85 °C for 10 years. Isolation was carried out using a modified Zymo Research Quick-RNA kit protocol. This isolation method showed significant improvement in RNA integrity when compared to RNA extracted using the original Boom method. RNA Sequencing provided high-quality data comparable to that of other studies using recently frozen blood in RNA stabilisation tubes. Additionally, sequencing data from blood collected in citrate and EDTA anticoagulants also showed excellent correlation.

**Supplementary Information:**

The online version contains supplementary material available at 10.1038/s41598-024-80287-4.

## Introduction

Ribonucleic acid (RNA) is a fundamental molecule involved in protein synthesis, gene regulation and cellular functions^1^, which serves as a cornerstone for various techniques aimed at unravelling biological complexities, in both diagnostic and research settings^2,3^. RNA is inherently unstable and susceptible to environmental and enzymatic degradation in-vitro, due to fluctuations in temperature and pH, and the presence of ubiquitous ribonucleases (RNases) that cleave RNA phosphodiester bonds^4,5^. Consequently, preserving RNA integrity during sample storage and biobanking is essential for subsequent procedures and analyses including RNA isolation, RNA sequencing (RNA-Seq) and reverse transcription polymerase chain reaction (RT-PCR), which rely on good-quality RNA for insights into gene expression patterns and disease biomarkers^6^. For larger collections, added benefits of the biobanking method would include affordability, minimal blood volume requirements and also preservation of RNA integrity during long-term storage^7^. Addressing these issues is critical for maximizing RNA’s utility in research and diagnostics and facilitating advancements in these areas.

In this article we report a method for isolating high-quality RNA that is suitable for RNA-Seq. RNA was isolated from biobanked citrated and ethylenediaminetetraacetic acid (EDTA) whole blood in lysis buffer as described in the Boom RNA isolation protocol^8^, thus avoiding the use of costly RNA stabilising blood tubes. All whole blood in lysis buffer samples were stored at −85 °C for 10 years and were collected as part of the Maltese Acute Myocardial Infarction (MAMI) study^9^.

## Results

### RNA yield and RIN using the original Boom’s RNA isolation^8^ method and the modified protocol of the Quick-RNA Whole Blood kit by Zymo Research

RNA isolated from citrated and EDTA whole blood in Boom’s lysis buffer^8^ using the original Boom isolation method^8^ gave mean yield of 1069 ng and 1031 ng, and mean RNA integrity number (RIN) of 4.8 and 4.1 respectively. DNA carryover was observed in electropherograms when using this method. RNA isolated from citrated and EDTA whole blood in lysis buffer using the modified Quick-RNA kit protocol described here gave mean yields of 1409 ng and 1223 ng, and excellent mean RINs of 8.4 and 8.7 respectively. No DNA carryover was observed in electropherograms (Table 1).

**Table 1:** Mean yield, mean RNA integrity number (RIN) and typical electropherogram for RNA from citrated and EDTA blood extracted using the original Boom isolation method and the modified Quick-RNA kit protocol described here.

	Original Boom & Sol’s Method		Modified Quick-RNA kit Method	
Anticoagulant	Citrate	EDTA	Citrate	EDTA
Number of samples	2	2	10	14
RNA yield mean & range (ng) Qubit: RNA BR Assay	1069 (819–1318)	1031 (768−1293)	1409 (721–2754)	1223 (313–2430)
RIN mean & range ScreenTape analysis kit	4.8 (4.7–4.9)	4.1 (3.8–4.3)	8.4 (8.0–8.6)	8.7 (7.9–9.6)
Typical electropherogram				

The RIN is higher with the modified Quick-RNA protocol. DNA carryover is evident as a peak on the far right in the electropherogram of the Boom method.

### RNA-Seq data quality assessment and comparison to data from recently-frozen blood

**Fig. 1:**
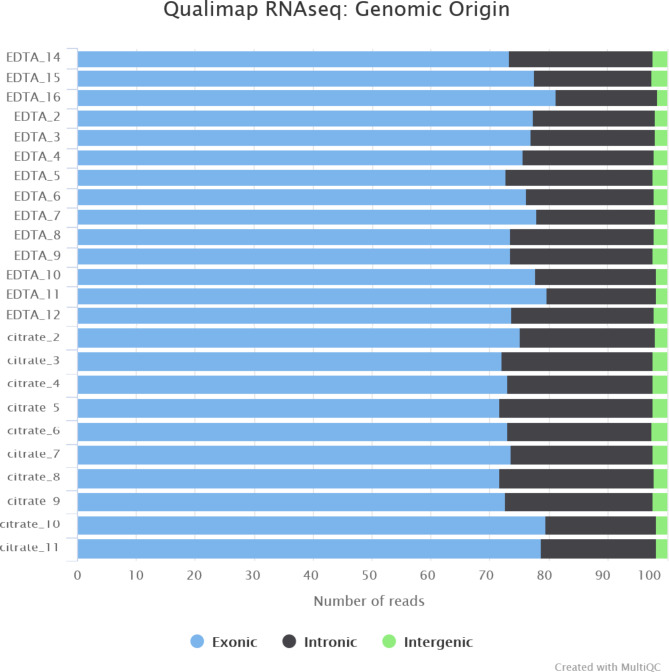
Percentages of exonic, intronic and intergenic regions in EDTA and citrated samples. Distribution of reads to genomic regions shows a low proportion of reads from intergenic regions, excluding major DNA contamination in both EDTA and citrated samples.

The overall quality of the RNA-Seq data from the RNA isolated using the modified Quick-RNA protocol was excellent, with an average Phred score of 35, even for the 3’ tail. Contamination was not an issue in the protocol since RNASeq was successful. No biological contamination was detected with FastQ Screen^10^. The RNA-Seq datasets were found to have a very low proportion of intergenic regions, ruling out DNA contamination (Fig. 1).

Upon gene quantification using featureCounts, at least 16,633 annotated genes were captured from each sample, after discarding genes with less than 10 raw counts observed in any sample. The median percentage of discarded genes with less than 10 raw counts were 13.72% (range 6.70−20.53%) for citrated samples and 14.24% (range 6.58–21.08%) for EDTA samples. The means were similar, at 14.31% and 14.36% respectively.

Additional QC parameters including mapping rates, insert sizes and duplication rates are shown in Supplementary Table 1, and they are as expected for RNA-Seq experiments.

**Fig. 2:**
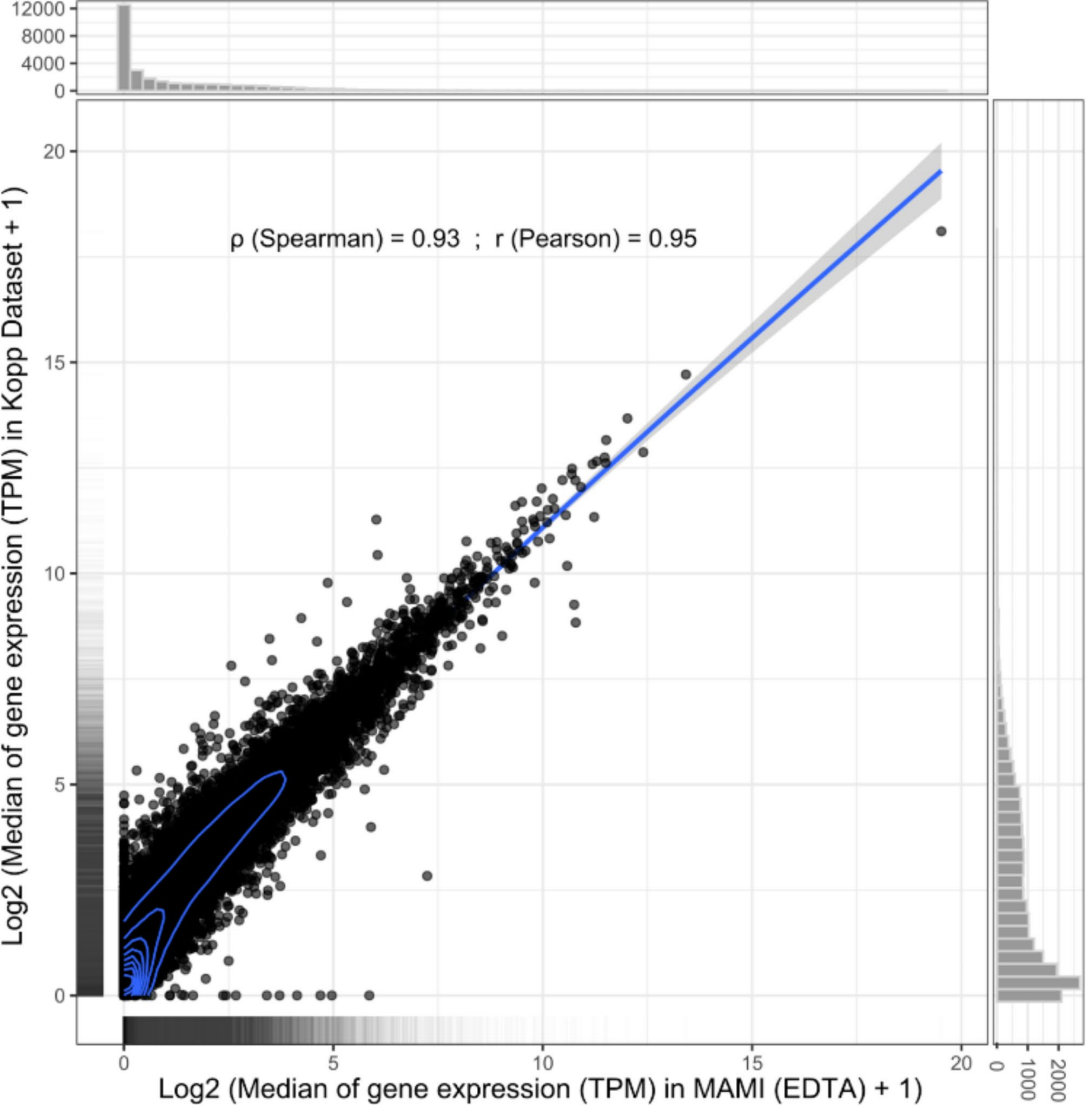
Good correlation in the median gene expression in Transcript per Million (TPM) was observed between the RNA-Seq data collected from RNA isolated from 10-year-old blood samples and data from healthy controls frozen for 1–2 years from the Kopp public dataset.

Aligned and quantified RNA-Seq data from ten EDTA blood samples was also compared to RNA-Seq data obtained from whole blood samples from twenty healthy controls frozen for 1–2 years (B Kopp 2022, pers. comm. 19 May) from the Kopp public dataset on cystic fibrosis^11^. Library preparation for both datasets was carried out using the TruSeq Stranded Total RNA with Ribo-Zero Globin Human kit (Illumina), with 150 base pair (bp) paired end reads. Both datasets gave very similar quality control results, including similar duplication rates and similar per sequence GC content. A good correlation in median gene expression was also observed between the two datasets, with a Spearman’s correlation of 0.93 and a Pearson’s correlation of 0.95 (Fig. 2).

### Comparison of data from EDTA and citrated whole blood

**Fig. 3:**
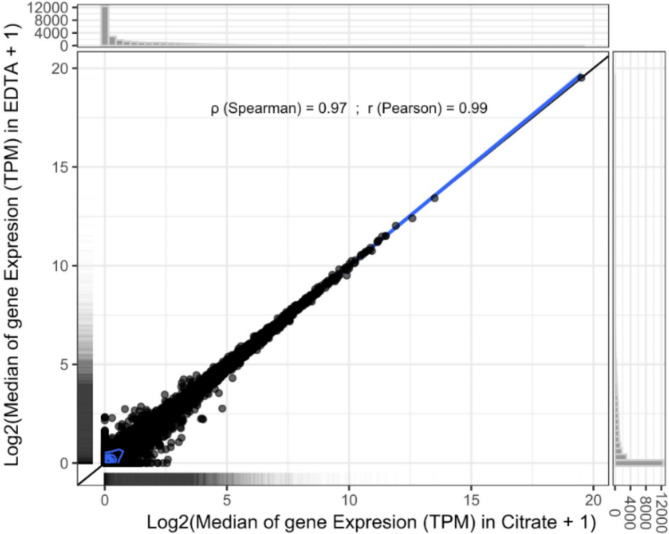
Good correlation in the median gene expression was observed between data from EDTA and citrated whole blood.

**Fig. 4:**
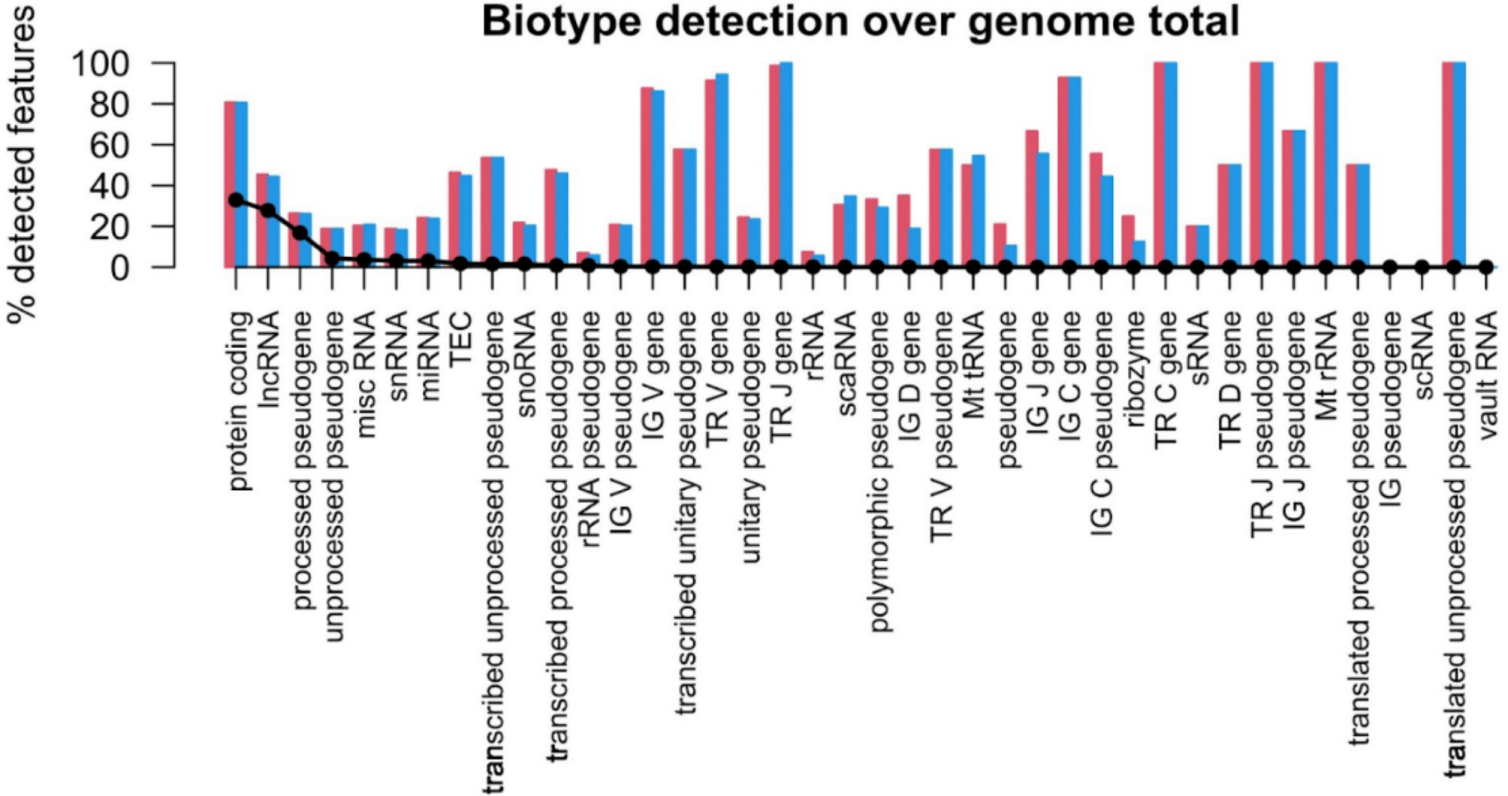
Comparison plot (produced with NOISeq v.2.40^12,13^) showing the percentage of each biotype in the genome detected from samples extracted with EDTA (blue) and citrated whole blood (pink). The black line displays the percentage abundance of each biotype within the genome. Abbreviations described in http://feb2021.archive.ensembl.org/info/genome/genebuild/biotypes.html.

An excellent Spearman’s and Pearson’s correlation in median gene expression of 0.97 and 0.99 respectively, was observed between RNA-Seq data from RNA isolated from ten citrated and ten EDTA whole blood samples from the same ten participants (Fig. 3). We also observe similar proportions of RNA biotypes between the two datasets (Fig. 4).

Since SNV information may also be useful from RNA-Seq data, a comparison between WGS data and RNA-Seq data from citrated and EDTA blood tubes was conducted. The genotype concordance between these was calculated across a panel of over 500 high-quality genomic SNVs with a minor allele frequency (MAF) > 0.6 in gnomAD genomes v3.1.2. For this analysis, only sites with a coverage of ≥ 30x in the RNA data and ≥ 20x in the DNA data were included, ensuring that at least 200 SNVs were assessed for each sample. Average percentage concordance in genotypes called between RNA-Seq and WGS genotype data for at least 200 SNVs was similar for citrated and EDTA samples, namely 88.7% (range 85.1–92.5%, *N* = 10) for the citrated samples and 89.0% (range 84.3–92.4%, *N* = 14) for the EDTA samples. Any non-concordance was for heterozygous genotypes which is expected due to bias in allelic expression. Unfortunately, the RNA extracted using the original Boom RNA isolation method had too poor a RIN score to qualify for RNA sequencing, and therefore no concordance test between the methods could be carried out.

## Discussion

Multi-omic studies herald a transformative era in biomedical research and diagnostics, as they integrate data from various molecular levels, and offer unprecedented opportunities to unravel the intricacies of biological systems with unparalleled depth and breadth^14^. At the heart of this multidimensional approach lies transcriptomics, which occupies a central position in deciphering the intricate interplay between genes, transcripts and proteins^15^. As technologies continue to evolve, transcriptomics is expected to play an increasingly pivotal role in understanding the complexities of cellular processes, disease mechanisms, and therapeutic interventions^16–18^. From uncovering novel biomarkers for early disease detection to elucidating gene regulatory networks, the future of transcriptomics holds vast potential to drive innovations in personalised medicine and precision healthcare^19,20^.

High-quality RNA serves as the foundation for all aspects of transcriptomics, which relies heavily on undegraded RNA to provide reliable and meaningful insights into the dynamic landscape of gene expression patterns. Maintaining quality is especially important when considering RNA’s innate susceptibility to degradation^4^. Degraded RNA leads to skewed results, false positives, and compromised data integrity, hindering the interpretation of experimental findings and undermining the validity of downstream analyses^21^. Therefore, meticulous attention to RNA quality control measures, from sample collection and preservation to RNA isolation, is paramount, especially for large sample collections and long-term storage scenarios.

The scope of our article is to provide a protocol for RNA isolation, which has been validated for long-term RNA storage and has shown success even for RNA sequencing applications. By focusing on this isolation protocol, we aim to provide researchers with a well-tested method for reliable RNA preservation over extended periods, as there is a growing demand for such protocols in the field. Other recently published protocols, like that of Nguyen et al.^22^, have demonstrated effective RNA extraction from frozen EDTA blood; however, their protocol was limited to blood samples stored for only 2–3 months.

In this study, RNA was isolated from 400 µL of whole blood in Boom’s lysis buffer^8^ that was frozen at −85 °C for 10 years. The resulting RNA yield and RIN were suitable for RNA-Seq library preparation, which usually require at least 500 ng and RIN > 7. Besides preserving RNA for many years, this method is also cost-effective and suitable for large collections. One of the primary drawbacks associated with RNA stabilisation tubes is their considerable cost. While these tubes offer the advantage of preserving RNA integrity during sample collection and storage, their procurement can inflate the overall expenses of research projects during the initial research subject recruitment phase, particularly when dealing with large sample sizes or long-term studies. RNA stabilisation tubes also require a specific draw volume, which is usually a relatively large sample volume of at least 2.5 mL. The volume of 400 µL of whole blood used as a starting material in this study makes the protocol feasible for multi-omic studies where many different sample types are required from the same blood sampling, as well as for studies with limited sample availability.

RNA from a total of 400 µL of citrated and EDTA whole blood in Boom’s lysis buffer^8^ was isolated using two different protocols: the Boom RNA isolation method^8^, and a modified protocol of the Zymo Research Quick-RNA Whole Blood kit. The Boom method is based on the principle of adsorption of nucleic acids onto silica particles in the presence of high concentrations of chaotropic salts. Following binding, the nucleic acids are washed to remove contaminants, and then eluted in a low-salt buffer^8^. This method gave good RNA yield but very poor RNA integrity, indicating RNA degradation during the isolation process which might be caused by the large amount of vortexing in the protocol. This protocol also does not include a deoxyribonuclease DNase step, which resulted in the presence of contaminating DNA in the final elution. The Boom method was noted to be quite labour-intensive and time-consuming, and provided limited scalability when compared to silica-based kits such as the Quick-RNA Whole Blood kit.

A high yield of RNA with excellent integrity that was suitable for RNA-Seq was extracted from both citrated and EDTA whole blood using the modified Zymo Research Quick-RNA kit protocol. The modifications that we applied included the addition of several centrifugation steps aiming to optimise the isolation protocol to increase RNA yield. An initial centrifugation step aided sample flow through the silica filter via a vacuum manifold by pelleting any cell debris. Recovery and elution centrifugation steps were also doubled to maximise the RNA yield. The RNA isolation kit protocol also offers several advantages over traditional RNA isolation methods including convenience, consistency and scalability, making it suitable for high-throughput applications. The method allows for the possibility of a DNase step which we included to eliminate DNA contamination. It is of note that this RNA isolation protocol requires that samples are stored in Boom’s lysis buffer.

The median gene expression from RNA isolated using the modified kit protocol was found to be comparable to that obtained from whole blood samples from the Kopp study^11^, which were stored in RNA stabilisation tubes and frozen for up to two years. This finding is indicative of the robustness and long-term stability of the RNA samples obtained through the modified kit protocol, affirming its suitability for preserving RNA integrity for at least one decade. Moreover, it highlights the feasibility of utilising this protocol as a cost-effective alternative to RNA stabilisation tubes for long-term storage of RNA samples without compromising sample or data quality in downstream analyses.

An excellent correlation was also observed in the median gene expression of RNA isolated from citrated and EDTA whole blood. This correlation likely stems from the meticulous management of preanalytical variables, notably the immediate pipetting of blood into Boom’s lysis buffer at the phlebotomy location and subsequent freezing at −85 °C within one hour of sample collection. This robust correlation underscores the importance of stringent sample handling procedures in preserving RNA integrity and minimising variations introduced during sample processing. Additionally, it further validates the reliability and reproducibility of the standardised experimental protocol employed, affirming its suitability for accurate gene expression analyses across different sample types and conditions. Furthermore, multiple researchers in our lab have successfully utilised this protocol for RNA isolation and high throughput RNA sequencing of another 972 samples throughout a span of a year.

In conclusion, this protocol for long-term whole blood storage, coupled with the successful isolation of high-quality RNA after a decade, represents a significant advancement in biobanking. The ability to preserve RNA integrity over extended periods opens doors to new possibilities in longitudinal studies, transcriptomics, biobanking, and clinical applications, where sample stability is paramount. The consistently excellent quality of the isolated RNA underscores the robustness and reliability of this protocol, ultimately advancing our understanding of biological systems and facilitating the translation of research findings into clinical practice.

## Methods

All samples used were collected as part of the Maltese Acute Myocardial Infarction (MAMI) study^9^. Written informed consent was obtained from all research participants involved in this study. The study was carried out in accordance with relevant guidelines and regulations approved by the University of Malta Research Ethics Committee (UREC Ref No MD 32/2010, FHS031/2014, Ref No 15062020 5549 6231).

Several aliquots were prepared by adding 100 µL of EDTA or citrated whole blood to pre-pipetted 900 µL of Boom’s^8^ lysis buffer (120 g guanidine thiocyanate, 100 mL Tris hydrochloride, 22 mL 0.2 M EDTA pH 8.0, 2.6 g Triton-X-100) in Micronic tubes with TPE push caps and mixed by inversion on-site immediately after phlebotomy. The tubes were labelled with cryogenic labels. Whole blood aliquots in lysis buffer were transported to the laboratory in portable coolers pre-chilled at at −20 °C and frozen at −85 °C in Micronic racks within 1 h of blood collection.

Isolation of RNA was carried out ten years later from four pooled aliquots of whole blood and lysis buffer using two different silica-based methods: the original Boom^8^ method and the Quick-RNA Whole Blood kit (Zymo Research), both with modifications. The original Boom^8^ method was modified slightly based on previous work to include an extra 70% ethanol wash. Furthermore, a volume of 33 µL, instead of 40 µL, of silica suspension was used, and DNA was eluted in nuclease free water instead of low salt buffer. The Quick-RNA Whole Blood kit protocol was modified by including (1) an initial centrifugation step to pellet any cell debris which may hinder the passage of the large sample volume through the silica filter, (2) double recovery, and (3) double elution centrifugation steps to maximise the RNA yield.

**Fig. 5:**
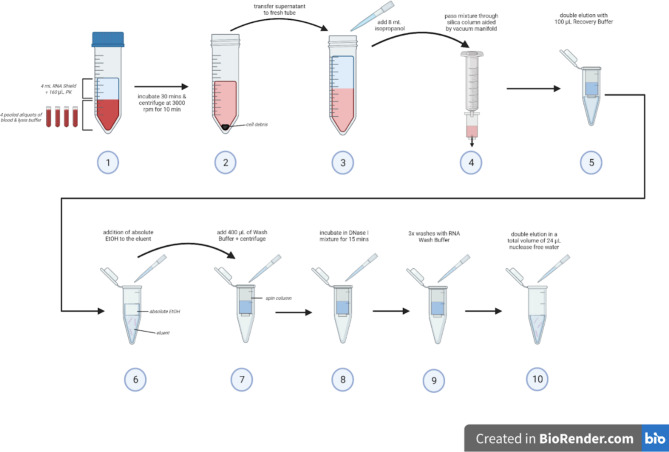
Summary of the modified protocol of the Zymo Research Quick-RNA Whole Blood kit.

The modified Quick-RNA Whole Blood kit protocol is summarised in Fig. 5 and a detailed description is as follows. A volume of 4 mL of DNA/RNA Shield™ and 160 µL of Proteinase K were added to four pooled thawed aliquots of blood in lysis buffer in a 50 mL sterile and RNase-free tube, and incubated at room temperature for 30 min. An initial centrifugation step was added to the kit protocol, whereby the mixture was centrifuged at 3000 rpm for 10 min. A volume of 8 mL of isopropanol was then added to the supernatant and mixed by vortexing. The mixture was then passed through a Zymo-Spin™ IIICG Column by the use of a vacuum pump and manifold. The column was placed on a collection tube and centrifuged at 16,000 g for 30 s at room temperature. All centrifugations after this step were carried out at this speed and for this amount of time. The protocol was modified slightly by including double recovery of the RNA. To elute the RNA from the column, 100 µL Recovery Buffer was added and the column centrifuged with the eluent recovered in a tube. This step was then repeated with another 100 µL Recovery Buffer which eluted into the same tube. An equal volume (200 µL) of absolute ethanol was added to the eluent. The RNA solution was then passed through the Zymo-Spin™ IC Column by centrifugation. The column was incubated with 40 µL of the kit DNase I mixture at room temperature for 15 min. A volume of 400 µL of RNA Prep Buffer was added followed by centrifugation. The flow-through was discarded and two washes were conducted, one with 700 µL of RNA Wash Buffer, and the second with 400 µL RNA Wash buffer, both followed by centrifugation and discarding of the flow-through. The column was transferred to a clean 1.5 mL tube. To improve RNA recovery, the single elution step was increased to two elutions in which 12 µL of nuclease-free water were added to the column which was then centrifuged. This step was repeated with another 12 µL water which was collected in the same 1.5 mL sterile and RNase free snap-lock tube. The tube was mixed by gentle flicking. A 2.5 µL aliquot was placed in a fresh 1.5 mL RNase-free tube for assessment of RNA quantity and quality which was carried out on 1 µL aliquots using Qubit™ RNA Broad Range kit (Thermo Fisher Scientific) and RNA ScreenTape kit (Agilent) respectively. This tube and the rest of the RNA were immediately frozen and kept at −85 °C until use.

The RNA samples were delivered to the sequencing service provider (Macrogen Europe, Netherlands) on dry ice with same-day delivery within 3 weeks of isolation. The samples were transported in 1.5 mL snap-lock tubes wrapped in parafilm. The tubes were transported in cryoboxes in a closed zip-lock plastic bag to minimise the evaporation of the sample during transportation.

Library preparation was carried out using the Illumina TruSeq Stranded Total RNA with Ribo-Zero Globin Human kit (catalogue number 20020597). This kit removes ribosomal RNA and globin mRNA. Sequencing was carried out on a NovaSeq 6000, with 150 bp paired-end reads, and 100 million reads per sample.

The quality of the RNA-Seq data obtained from the RNA samples isolated with the modified Quick-RNA Whole Blood was assessed using FastQC^23^/MultiQC^24^, where raw reads were examined for possible low base scores and contamination. Contamination screening was checked using FastQ Screen^10^ (v0.14.1). Quality control (QC) was followed by the trimming of Illumina adapters using Cutadapt^25^ (v2.8) through the Trim Galore! wrapper tool and removal of low complexity reads, trailing bases with quality below 30, and paired reads for which both mates did not reach a length of 70 bases using Prinseq + + ^26^ (v1.2). The data was aligned to Genome Reference Consortium Human (GRCh) Build 38 with Ensembl annotation version 103 using STAR^27^ (v2.7.3a). The distribution of reads to genomic regions (exonic, intronic and intergenic) was checked using Qualimap^28^ (v2.2.2-dev). After sorting the BAM files by name with Samtools (v1.10), the uniquely mapped fragments were used to compute gene quantification with featureCounts^29^ (v2.0.1).

## Electronic supplementary material

Below is the link to the electronic supplementary material.

Supplementary Material 1

## Data Availability

The dataset generated during the current study is available from the corresponding author on reasonable request and subject to ethics approval.
